# IL27 expression and IL27RA-dependent cellular responses are associated with biochemical recurrence and immune-regulatory features in prostate cancer

**DOI:** 10.3389/fimmu.2026.1931108

**Published:** 2026-08-10

**Authors:** Yuliang Huang, Yurui Tang, Cunhe Yuan, Jia Zhang

**Affiliations:** 1 Department of Urology, The Fourth Affiliated Hospital of Anhui Medical University, Chaohu, Anhui, China; 2 Ward 2 of Gastroenterology, Combined Ward of Rheumatology and Immunology, The Fourth Affiliated Hospital of Anhui Medical University, Chaohu, Anhui, China

**Keywords:** biochemical recurrence, IL-27, IL27RA, immune evasion, ligand–receptor signaling, prostate cancer, pyroptosis, tumor microenvironment

## Abstract

**Introduction:**

Biochemical recurrence remains a clinically heterogeneous event in prostate cancer, yet the immune signals associated with relapse-prone tumors are not fully defined. This study investigated IL-27 and its receptor IL27RA as a potential immune–epithelial ligand–receptor axis linking microenvironmental inflammation, recurrence, and immune escape.

**Methods:**

Transcriptomic and clinical datasets from TCGA and GEO were analyzed to assess IL-27 expression, biochemical recurrence-free survival, clinicopathological associations, immune infiltration, pathway activity, single-cell distribution, mutation features, and druggable-target patterns. Experimental validation was performed in RWPE-1 prostate epithelial cells and PC-3 prostate cancer cells using immunofluorescence staining, recombinant IL-27 stimulation, IL27RA knockdown, CCK-8 assays, and qRT-PCR.

**Results:**

Higher IL27 expression was observed in prostate cancer and was associated with recurrence, higher Gleason score, and shorter biochemical recurrence-free survival; the association with recurrence persisted after adjustment for the clinicopathological variables included in the Cox model.

**Discussion:**

These findings identify IL27 expression as a recurrence-associated molecular feature in retrospective prostate cancer datasets and provide proof-of-concept evidence for an IL27RA-dependent response to IL-27 in PC-3 cells.

## Introduction

Prostate cancer (PCa) remains one of the most prevalent malignancies in men and represents a major cause of cancer-related mortality worldwide (1, 2). Although localized disease can often be managed by radical prostatectomy, radiotherapy, or systemic treatment, a substantial proportion of patients still develop biochemical recurrence (BCR) during follow-up (3). BCR is clinically important because it may precede radiographic progression and often indicates a higher probability of subsequent treatment failure. Current risk assessment still relies largely on prostate-specific antigen kinetics, Gleason score, tumor stage, and other clinicopathological variables. However, patients with similar conventional risk profiles may show different recurrence trajectories, suggesting that additional molecular and microenvironmental determinants are involved in post-treatment disease reactivation.

Recent studies indicate that molecular information can improve the biological interpretation of recurrence risk. In prostate adenocarcinoma, cuproptosis-related immune signatures have been reported to reflect tumor microenvironmental features and prognostic differences (4). In other solid tumors, circulating tumor DNA mutation panels have also been explored for early relapse prediction and survival monitoring (5). These findings support the broader concept that recurrence is not only a clinical endpoint but also a molecularly traceable disease state. Therefore, immune-related molecules that link tumor microenvironmental remodeling with BCR may provide additional value for recurrence-oriented PCa stratification.

The tumor microenvironment communicates with malignant epithelial cells through cytokines, receptor pairs, stromal cues, and immune-cell-derived signals. Ligand–receptor signaling is particularly useful for studying this process because it connects a regulatory signal with a defined responding cell population. In PCa, this framework is clinically relevant because immunotherapy benefit has been observed mainly in selected molecular settings rather than in unselected disease, as illustrated by reported activity of pembrolizumab in MSI-high or BRCA-positive castration-resistant PCa (6).

Interleukin-27 (IL-27) is an IL-12-family cytokine formed by Epstein–Barr virus-induced gene 3 and p28 (7, 8). It is mainly associated with antigen-presenting and myeloid-cell responses, including dendritic cell and macrophage activity under inflammatory conditions (9, 10). IL-27 signals through a receptor complex containing IL27RA/WSX-1 and gp130 and can activate JAK/STAT-related pathways (11). Through this receptor system, IL-27 participates in T-cell differentiation, cytotoxic lymphocyte activity, macrophage-associated regulation, and immune inhibitory programs.

The biological role of IL-27 in cancer is complex. On the one hand, IL-27 can support antitumor immunity by enhancing T-cell differentiation and cytotoxic immune responses. On the other hand, IL-27 can also induce immunosuppressive mediators, including PD-L1 and IL-10, thereby contributing to immune restraint and tumor immune escape in certain contexts (12). This duality indicates that IL-27 should not be classified as uniformly antitumor or protumor. Instead, its function depends on tumor lineage, receptor expression, immune-cell composition, and the dominant signaling state within the tumor microenvironment.

Findings from different cancer types further support this context-dependent interpretation. In hepatocellular carcinoma, IL-27 receptor signaling has been reported to regulate innate cytotoxic lymphocytes in a checkpoint-like manner and to support tumor development across different disease etiologies (13). In melanoma, reduced CD40/IL-27 signaling has been associated with tumor-associated macrophage infiltration and resistance to immune checkpoint blockade (14). Conversely, IL-27 has also been reported to suppress melanoma cell proliferation through WSX-1/STAT1 signaling, and intratumoral delivery of lipid nanoparticles carrying IL-12 and IL-27 mRNA has shown antitumor activity in melanoma models (15, 16). IL-27-related signaling has additionally been linked to tumor-associated macrophages, regulatory T cells, and myeloid-derived suppressor cells, suggesting that its tumor effect may be strongly shaped by the immunological context (14, 17, 18).

Compared with these tumor types, the role of IL-27 in PCa remains insufficiently defined. Existing evidence suggests that IL-27 combined with cabozantinib may have therapeutic potential in bone metastatic PCa, partly through effects related to osteogenic remodeling (19). However, this does not clarify whether endogenous IL-27 expression is associated with biochemical recurrence, whether IL-27 marks an immune-evasive microenvironment, or whether IL-27 can act directly through IL27RA to modify prostate cancer cell phenotypes. These unresolved questions are relevant because immune responsiveness in advanced PCa appears to depend on defined molecular and immune features, as illustrated by the activity of pembrolizumab in selected MSI-high or BRCA-positive castration-resistant PCa settings.

Based on these considerations, this study investigated IL-27 as a potential immune–parenchymal ligand involved in recurrence-prone PCa. We integrated transcriptomic datasets from TCGA and GEO to evaluate IL-27 expression, BCR-free survival, clinicopathological associations, immune infiltration, functional pathways, somatic mutation features, and predicted drug sensitivity. Single-cell RNA sequencing was used to determine the cellular distribution of IL-27 and to assess whether IL-27-enriched cellular states were associated with recurrence outcomes. Finally, RWPE-1 cells and the PC-3 prostate cancer cell line were used for focused validation of the relationship between IL-27 exposure, IL27RA expression, cell viability, and immune-regulatory gene expression. This integrated analytical strategy follows the broader rationale that computational biomarker discovery can help prioritize biologically meaningful immune-related targets for subsequent experimental validation (20).

## Materials and methods

### Data collection and preprocessing

Transcriptomic matrices, mutation files, and clinical annotations were retrieved from TCGA and GEO. TCGA expression values were analyzed after log2(TPM + 1) transformation. Cases were retained only when IL27 expression and recurrence-related information were available; samples lacking essential clinicopathological variables were excluded from analyses requiring those variables. For prostate cancer cohorts, BCR status, recurrence-free survival time, Gleason score, tumor stage, and other available clinical factors were extracted. In computational analyses, IL27 refers to the transcript encoding the p28 subunit of IL-27, whereas IL-27 denotes the recombinant cytokine or the protein detected by immunofluorescence in cell-based experiments. IL27 transcript abundance alone does not establish formation of the p28–EBI3 heterodimer or activation of the IL27RA–IL6ST/gp130 receptor complex.

### IL-27 grouping and differential expression analysis

Patients were stratified by the median IL27 expression value. Differentially expressed genes between IL-27-high and IL-27-low tumors were identified using the limma package and visualized by heatmap and volcano plot. These gene sets were then analyzed using clusterProfiler for Gene Ontology and KEGG annotation. Gene Set Enrichment Analysis was applied to examine pathway-level differences between the two IL-27-defined groups. Immune regulation, cytokine signaling, NF-κB activation, and pyroptosis-related terms were retained for focused interpretation because they were directly relevant to inflammatory and immune-evasive tumor states.

### Survival analysis and prognostic nomogram

Kaplan–Meier analysis was used to compare biochemical recurrence-free survival between patients with high and low IL27 expression, using the median expression value as the cut-off. Survival differences were assessed with the log-rank test. The Kaplan–Meier analysis included 497 patients (249 IL27-low and 248 IL27-high), and 92 biochemical recurrence events were recorded. IL27 expression was entered as a continuous variable in the multivariable Cox proportional hazards model, which adjusted for tumor T stage and primary Gleason grade. T2 and primary Gleason grade 3 served as the reference categories. Hazard ratios were reported with 95% confidence intervals. An exploratory nomogram was constructed from IL27 expression, tumor T stage, and primary Gleason grade. Calibration at 1, 3, and 5 years was evaluated using 1, 000 bootstrap resamples in the complete-case cohort (n = 491).

### sc RNA-sequencing analysis

Single-cell RNA-sequencing data from six patients with castration-resistant prostate cancer in GSE137829 were analyzed using Seurat. The original study generated the libraries with the 10x Genomics platform and sequenced them on an Illumina NovaSeq 6000. The processed gene-by-cell expression matrices deposited in GEO were used for the present analysis. Retained cells underwent normalization, scaling, dimensionality reduction, and clustering. Major cell compartments were annotated according to canonical marker genes and cluster-enriched expression patterns. UMAP plots were used to display cell distribution and IL27 transcript detection. Epithelial cells were extracted and reclustered to resolve IL27-enriched epithelial subpopulations, and SeuratExtend was used for visualization. Because IL27 was sparsely detected at the single-cell level, a zero count in an individual cell was not interpreted as definitive absence of expression. Recurrence-free survival analysis was conducted at the patient level after consensus clustering and did not treat individual cells as independent survival observations.

### Immune infiltration analysis

Immune-cell composition was inferred using CIBERSORT and summarized with IOBR. Associations between IL27 expression, immune-cell estimates, and selected immune-related genes were calculated by correlation analysis and visualized with corrplot. Immune score, stromal score, and ESTIMATE score were generated with the ESTIMATE package to evaluate microenvironmental composition. Somatic mutation files were analyzed with maftools. Variant classes, single-nucleotide variant patterns, recurrently mutated genes, oncoplots, and co-mutation relationships were compared between IL-27-high and IL-27-low tumors. Druggable or actionable alteration categories were summarized separately to provide targetability-related genomic context.

### Consensus clustering

Unsupervised consensus clustering was performed using the ConsensusClusterPlus R package. The partitioning around medoids algorithm was applied, and candidate cluster numbers from k = 2 to k = 7 were tested. The optimal clustering solution was selected according to clustering stability and consensus matrix patterns. The resulting clusters were used for subgroup comparison and recurrence-free survival analysis.

### Cell culture and IL-27 treatment

RWPE-1 normal prostate epithelial cells and PC-3 prostate cancer cells were obtained from Procell Life Science & Technology Co., Ltd. Cells were cultured in supplier-recommended complete media at 37 °C with 5% CO_2_ and used during logarithmic growth. RWPE-1 cells were included as a non-malignant epithelial reference for immunofluorescence staining, whereas all cytokine-stimulation, IL27RA-silencing, viability, and gene-expression experiments were performed in PC-3 cells. For cytokine stimulation, PC-3 cells received recombinant human IL-27 at 0, 25, 50, or 100 ng/mL. The dose-screening assay identified 50 ng/mL as the working concentration for subsequent experiments.

### Immunofluorescence staining

Cells grown on coverslips were fixed, permeabilized, blocked, and incubated overnight with Abcam antibodies against IL-27 or IL27RA. Fluorescent secondary antibodies and DAPI were then applied. Images were acquired under identical exposure settings, and the scale bar was 10 μm.

### siRNA-mediated IL27RA knockdown

PC-3 cells were transfected with IL27RA-targeting siRNA or negative control siRNA using Lipo3000. Medium was replaced 6–8 h after transfection, and cells were collected 24–48 h later. For receptor-silencing experiments under cytokine stimulation, si-IL27RA-transfected cells were treated with recombinant IL-27 before qRT-PCR or viability analysis. Knockdown efficiency was confirmed by qRT-PCR.

### Cell viability assay

Cell viability was measured using CCK-8. For dose screening, PC-3 cells were exposed to 0, 25, 50, or 100 ng/mL recombinant IL-27 and measured at 0, 12, 24, 36, and 48 h. For functional validation, control, IL-27, si-IL27RA, and IL-27 plus si-IL27RA groups were compared. Absorbance was recorded at 450 nm after CCK-8 incubation. All CCK-8 experiments were performed in three independent biological replicates.

### Quantitative real-time PCR

Total RNA from PC-3 cells was reverse-transcribed and analyzed using SYBR Green-based qRT-PCR. IL27RA, CD274, IDO1, and PDCD1LG2 were measured to assess the transcriptional response to IL-27 and the expression of selected immune-regulatory genes. β-actin was used for normalization, and relative expression was calculated using the 2^-ΔΔCt method. All qRT-PCR experiments were performed in three independent biological replicates.

### Statistical analysis

Statistical analyses were performed using R software version 4.1.3. Continuous variables were compared using the Wilcoxon rank-sum test or Kruskal–Wallis test. Categorical variables were analyzed using the chi-squared test or Fisher’s exact test according to data distribution and sample size. Survival differences were assessed using Kaplan–Meier curves and log-rank tests, and multivariable associations were evaluated using Cox proportional hazards models. For differential-expression and functional-enrichment analyses, p values were adjusted for multiple testing using the Benjamini–Hochberg method, and adjusted p < 0.05 was considered statistically significant. Other analyses were exploratory and used two-sided nominal p values < 0.05 unless otherwise specified. Hazard ratios are reported with 95% confidence intervals.

## Results

### IL-27 expression is associated with biochemical recurrence in prostate cancer

**Figure 1:**
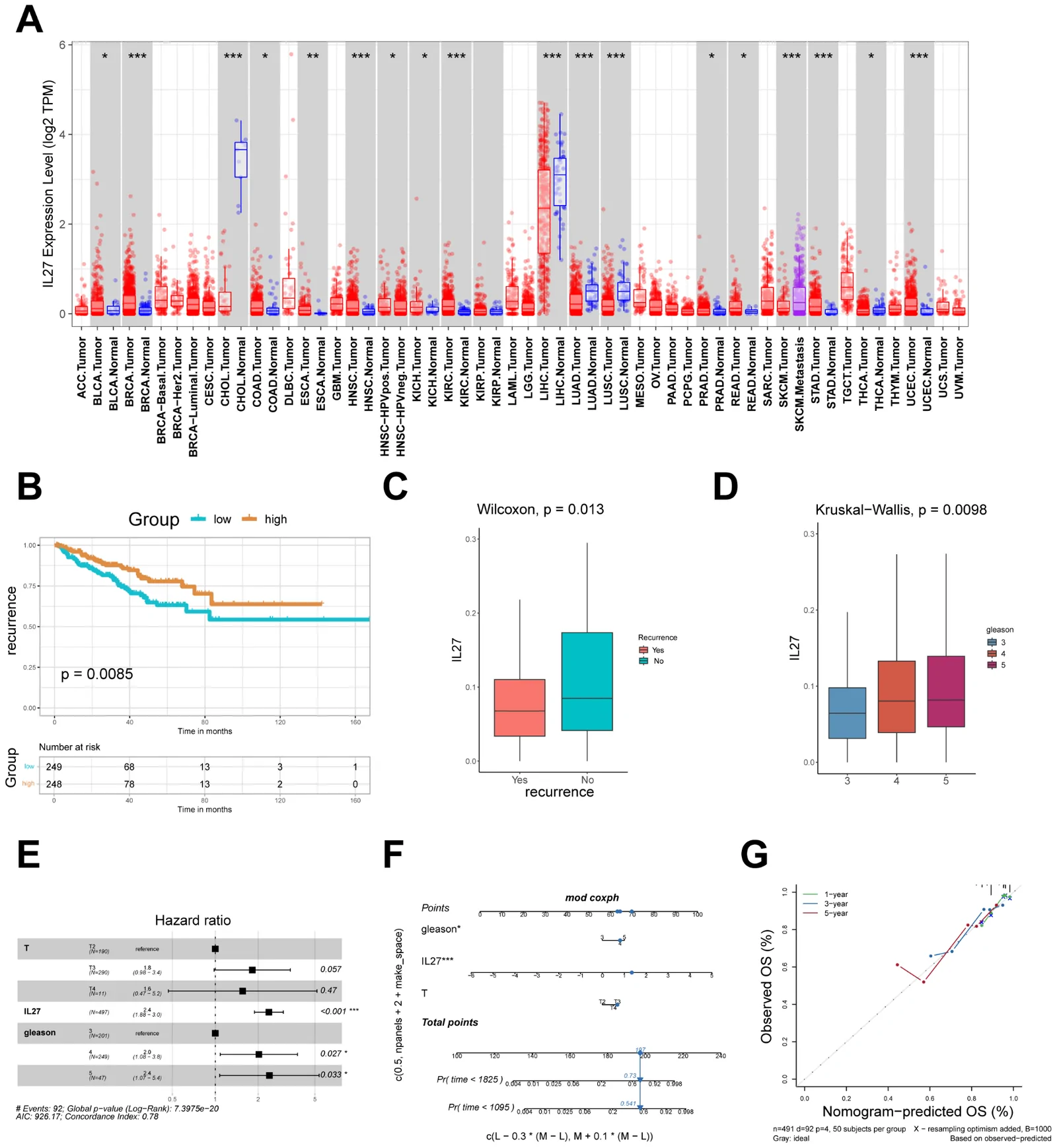
IL-27 expression and clinical relevance in prostate cancer. **(A)** IL-27 expression across tumor and matched normal tissues in multiple cancer types. **(B)** Kaplan–Meier analysis of biochemical recurrence-free survival using the median IL27 expression value as the cut-off (n = 497; IL27-low, n = 249; IL27-high, n = 248; log-rank p = 0.0085). **(C)** Comparison of IL-27 expression between patients with and without recurrence. **(D)** IL-27 expression across different Gleason score groups in prostate cancer. **(E)** Multivariable Cox proportional hazards model including continuous IL27 expression, tumor T stage, and primary Gleason grade. Squares indicate hazard ratios, and horizontal lines indicate 95% confidence intervals; 92 biochemical recurrence events were included. **(F)** Nomogram integrating IL-27 expression and clinical parameters, including Gleason score, for recurrence prediction. **(G)** Bootstrap calibration of predicted and observed biochemical recurrence probabilities at 1, 3, and 5 years using 1, 000 resamples in the complete-case cohort (n = 491). The diagonal line indicates ideal calibration.

We first asked whether IL27 expression differed between malignant and non-malignant prostate tissues and whether this difference was clinically informative. IL27 expression was higher in tumor tissues than in normal tissues (**Figure 1A**). When patients were stratified by IL27 expression, the IL-27-high group showed shorter recurrence-free survival than the IL-27-low group (p = 0.0085; **Figure 1B**). IL27 expression was also increased in recurrent cases (p = 0.013; **Figure 1C**) and rose with increasing Gleason score (p = 0.0098; **Figure 1D**).

In the multivariable Cox model, higher IL27 expression remained associated with biochemical recurrence after adjustment for tumor T stage and primary Gleason grade (HR, 2.40; 95% CI, 1.88–3.00; p < 0.001; **Figure 1E**). Relative to T2 disease, T3 stage showed a borderline association with recurrence (HR, 1.80; 95% CI, 0.98–3.40; p = 0.057), whereas T4 stage was not associated with recurrence (HR, 1.60; 95% CI, 0.47–5.20; p = 0.47). Primary Gleason grades 4 and 5 were associated with higher recurrence risk than grade 3 (grade 4: HR, 2.00; 95% CI, 1.08–3.80; p = 0.027; grade 5: HR, 2.40; 95% CI, 1.07–5.40; p = 0.033). An exploratory nomogram integrating IL27 expression, tumor T stage, and primary Gleason grade was constructed to estimate biochemical recurrence risk at 1, 3, and 5 years (**Figure 1F**). The model had a concordance index of 0.78, and bootstrap calibration compared the predicted and observed recurrence probabilities at the corresponding time points (**Figure 1G**). These analyses support an association between IL27 expression and recurrence risk, but the model requires validation in an independent cohort.

### IL-27-high tumors show distinct transcriptional features

**Figure 2:**
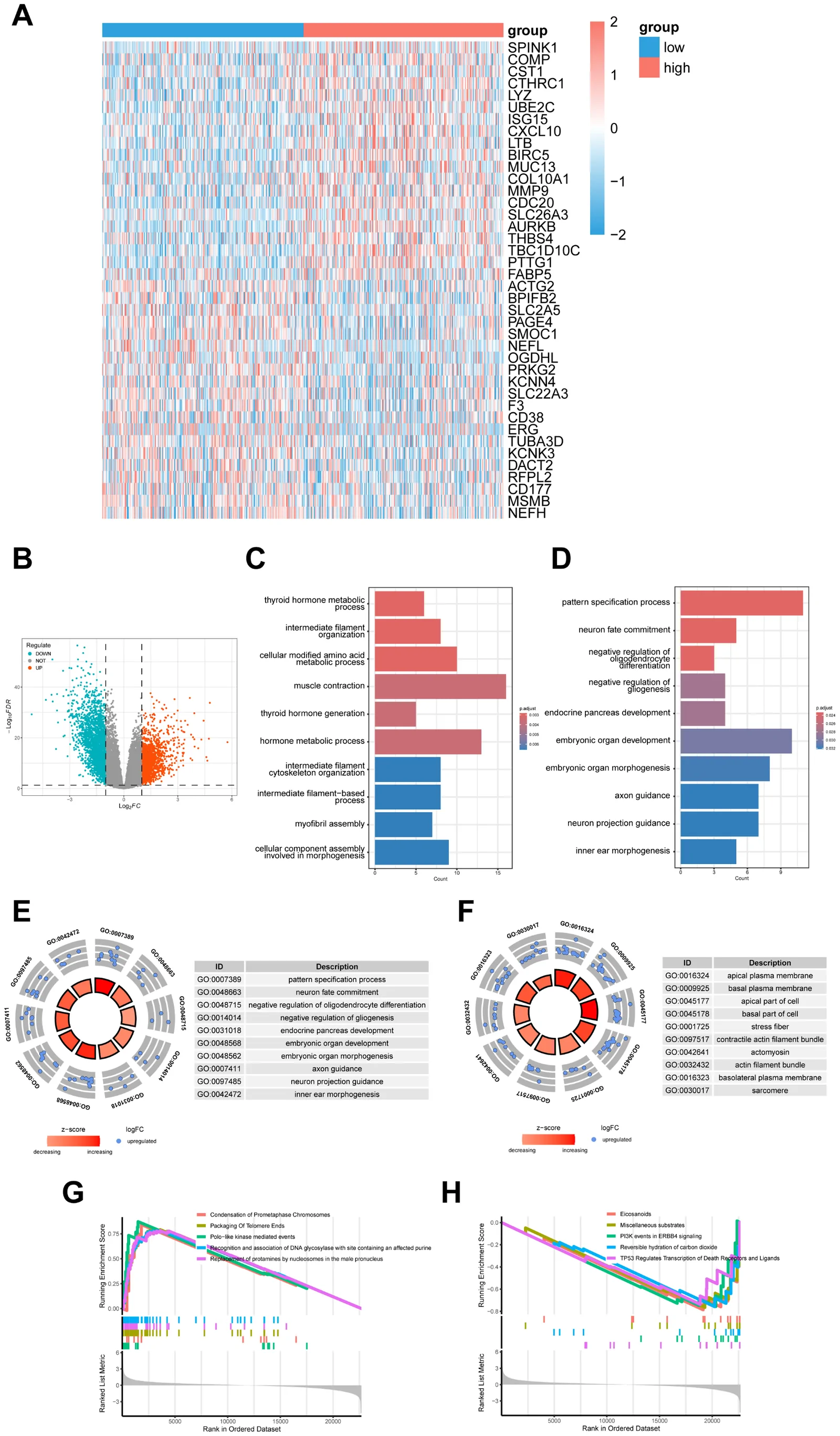
Transcriptional differences and functional enrichment associated with IL-27 expression. **(A)** Heatmap of differentially expressed genes between IL-27-high and IL-27-low prostate cancer samples. Rows indicate genes, and columns indicate samples. Normalized expression values are color-coded from low to high expression. **(B)** Volcano plot of differentially expressed genes between the two IL-27 expression groups. Significantly upregulated and downregulated genes are highlighted according to log2 fold-change and adjusted p-value thresholds. **(C)** Gene Ontology enrichment analysis of biological processes among genes upregulated in the IL-27-high group. **(D)** Gene Ontology cellular component enrichment analysis of genes downregulated in the IL-27-high group. **(E)** Circular plot summarizing enriched Gene Ontology cellular component terms among upregulated genes. **(F)** Circular plot summarizing enriched Gene Ontology terms among downregulated genes. **(G)** Gene Set Enrichment Analysis of pathways enriched in the IL-27-high group. **(H)** Gene Set Enrichment Analysis of pathways enriched in the IL-27-low group. *p < 0.05, ** p < 0.01, ***p < 0.001.

To define the molecular background associated with IL-27 expression, patients were divided into IL-27-high and IL-27-low groups. The heatmap showed clear separation of differentially expressed genes between the two groups, indicating that IL-27 expression corresponded to a distinct transcriptional state (**Figure 2A**). The volcano plot further confirmed that multiple genes were significantly upregulated or downregulated in IL-27-high tumors (**Figure 2B**).

Functional enrichment analysis was then used to characterize these transcriptional differences. Genes upregulated in the IL-27-high group were enriched in biological processes related to thyroid hormone metabolism, intermediate filament organization, muscle contraction, morphogenesis, and differentiation-associated programs (**Figures 2C, E**). By contrast, genes downregulated in the IL-27-high group were linked to neuron fate commitment, embryonic organ development, apical plasma membrane organization, and actomyosin-related structural components (**Figures 2D, F**).

Gene Set Enrichment Analysis provided an additional pathway-level view. IL-27-high tumors were enriched for programs such as prophase chromosome condensation and meiotic synapsis, whereas selected biosynthetic and immune-signaling pathways were more prominent in the IL-27-low group in this comparison (**Figures 2G, H**). Together, these data show that IL-27 expression is accompanied by broad transcriptional remodeling in prostate cancer.

### IL27-high tumors show transcriptome-inferred immune enrichment with suppressive immune features

**Figure 3:**
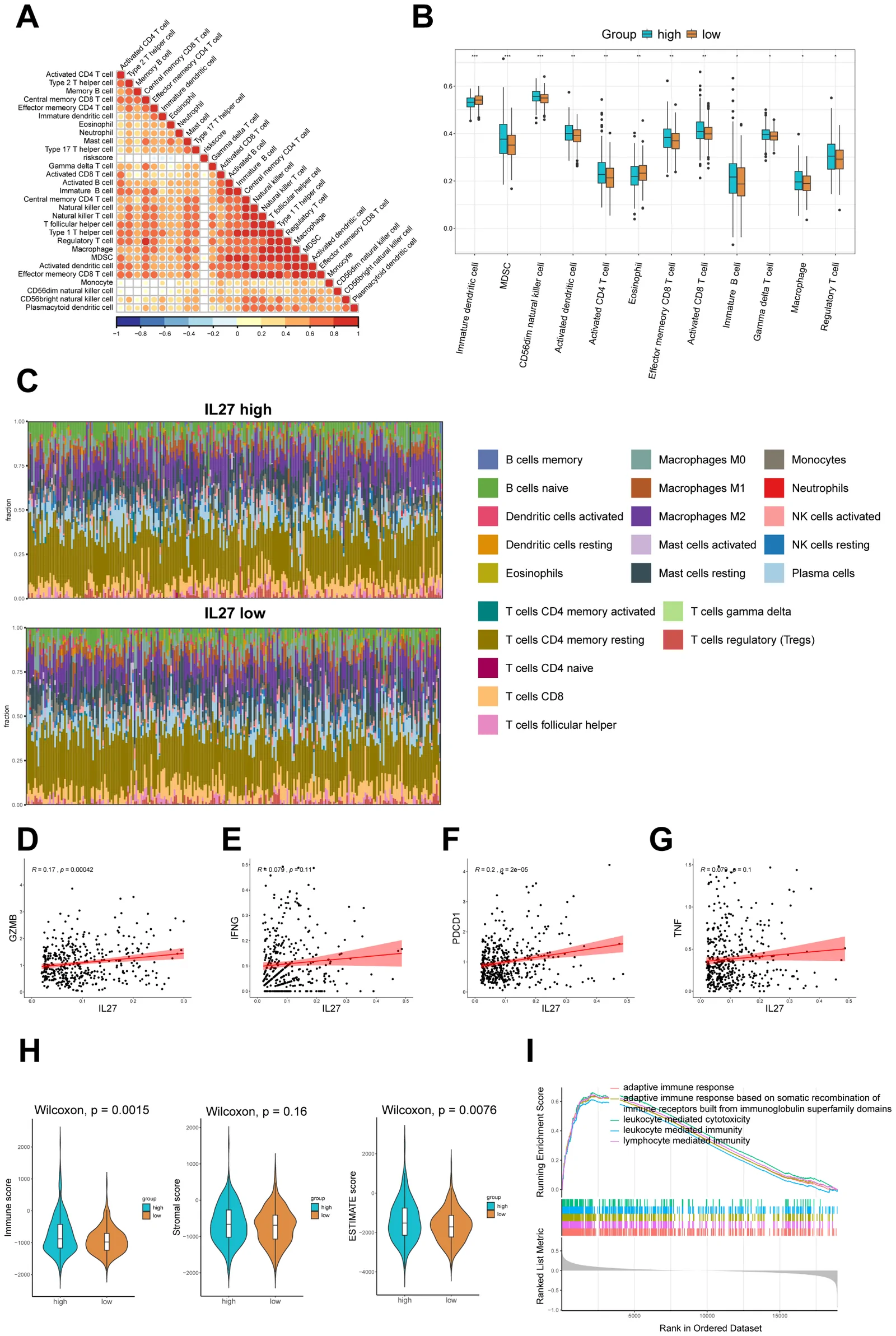
Association between IL27 expression and transcriptome-derived immune features in prostate cancer. **(A)** Correlation matrix of immune-cell infiltration across immune-cell subsets. Red indicates positive correlations, and blue indicates negative correlations. **(B)** Comparison of immune-cell infiltration between IL-27-high and IL-27-low groups. **(C)** Relative immune-cell composition in samples with high or low IL-27 expression. **(D–G)** Correlations between IL-27 expression and immune or inflammatory genes, including GZMB, IFNG, PDCD1, and TNF. **(H)** Immune score, stromal score, and ESTIMATE score in IL-27-high and IL-27-low groups. Statistical comparisons were performed using the Wilcoxon test. **(I)** Gene Set Enrichment Analysis of immune-related pathways associated with high IL-27 expression. *p < 0.05, ** p < 0.01, ***p < 0.001.

We next examined whether IL-27 expression was related to immune-cell infiltration in the prostate cancer microenvironment. Correlation analysis revealed that IL-27 was associated with multiple immune-cell populations, with notable positive relationships involving immunosuppressive populations such as myeloid-derived suppressor cells and regulatory T cells (**Figure 3A**). Group-level comparison confirmed that these suppressive immune components were more abundant in IL-27-high tumors than in IL-27-low tumors (**Figure 3B**).

The immune-cell composition differed substantially between the two IL-27-defined groups, suggesting that IL-27 expression was linked to remodeling of the tumor immune microenvironment (**Figure 3C**). IL-27 expression also showed positive correlations with immune and inflammatory genes, including GZMB, IFNG, PDCD1, and TNF (**Figures 3D–G**). These associations indicate that IL-27-high tumors contain active immune and inflammatory signals rather than a simple immune-cold state.

Consistently, IL-27-high tumors had higher immune scores and ESTIMATE scores, reflecting increased immune and stromal components within the tumor microenvironment (**Figure 3H**). GSEA further connected IL-27 expression with adaptive immune response and lymphocyte-mediated immunity (**Figure 3I**). Together, these analyses support an association between IL27 expression and an immune-enriched, transcriptionally regulated tumor state. Because the immune-cell estimates were derived computationally, the results do not establish changes in tissue-level immune-cell density or spatial distribution.

### Single-cell analysis reveals cellular heterogeneity of IL-27 expression

**Figure 4:**
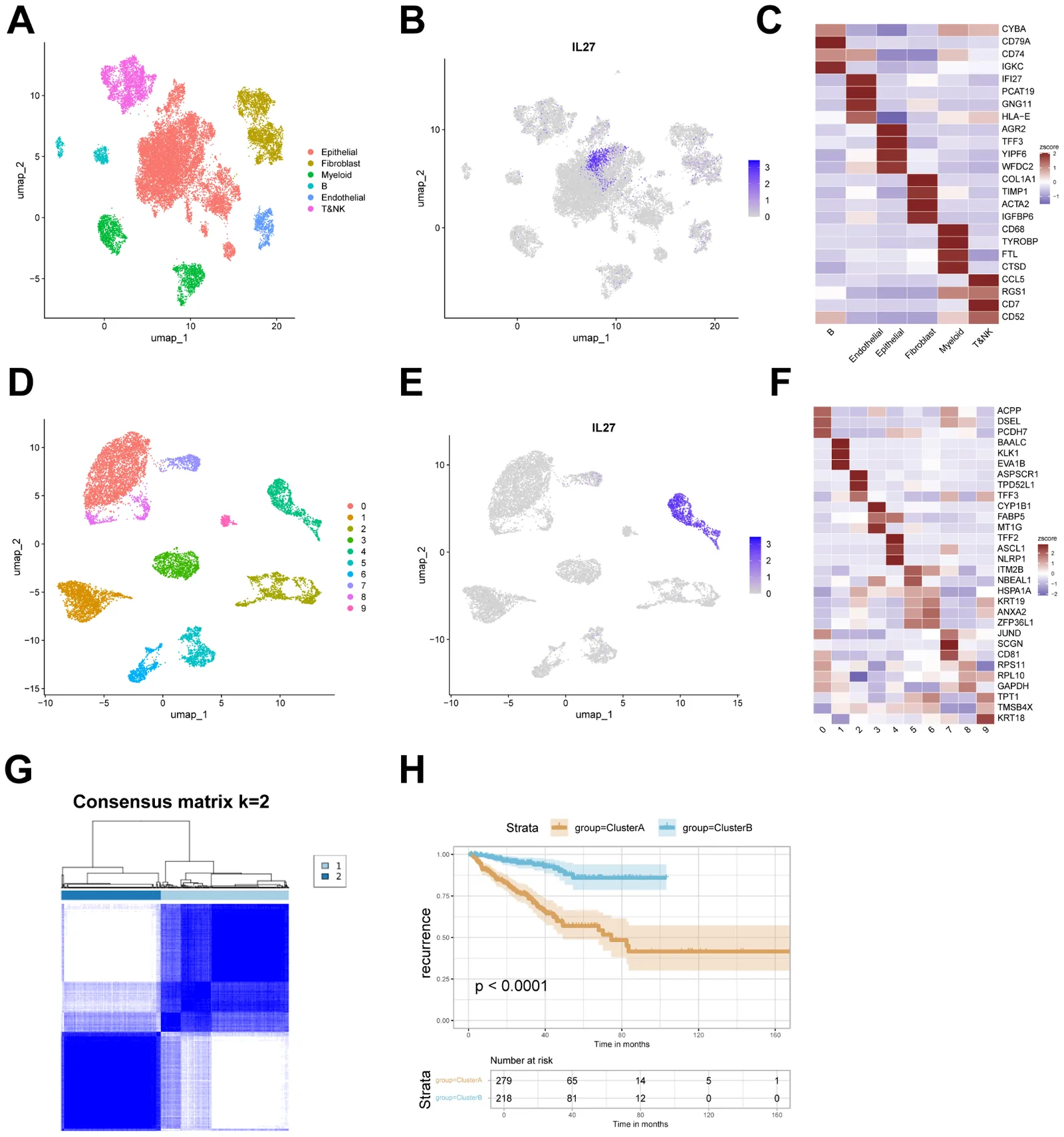
Single-cell analysis of IL-27 expression in the prostate cancer microenvironment. **(A)** UMAP visualization of major cell populations identified in prostate adenocarcinoma samples. **(B)** Distribution of IL-27 expression across all single cells. **(C)** Heatmap of representative marker genes used for cell-type annotation. **(D)** UMAP visualization of epithelial-cell subclusters after secondary clustering. **(E)** IL-27 expression across epithelial-cell subclusters. **(F)** Heatmap of differentially expressed genes among epithelial-cell subclusters. **(G)** Consensus matrix for unsupervised clustering at k = 2. **(H)** Kaplan–Meier recurrence-free survival analysis of the two consensus-defined groups.

Bulk transcriptomic analysis cannot determine which cellular compartments contribute to IL-27 expression. Therefore, we analyzed single-cell RNA-sequencing data to localize IL-27 within the prostate cancer microenvironment. UMAP clustering identified several major cell populations, including epithelial cells, fibroblasts, myeloid cells, endothelial cells, and T/NK cells (**Figure 4A**). IL-27 expression was not evenly distributed across these populations (**Figure 4D**) but was enriched in selected epithelial and immune-related clusters (**Figures 4B, E**).

Marker-gene heatmaps confirmed the identities of the annotated cell populations and showed distinct transcriptional profiles across cellular compartments (**Figures 4C, F**). Further subgroup analysis separated samples into two major clusters. Cluster B showed higher IL-27 expression than Cluster A (**Figure 4G**). Patients in Cluster B had shorter biochemical recurrence-free survival, suggesting that IL-27-enriched cellular states may have prognostic relevance (**Figure 4H**).

Consensus clustering supported k = 2 as a stable classification, confirming the robustness of this subgrouping strategy (**Supplementary Figure S1**). These findings indicate that IL-27 expression reflects cellular heterogeneity within the prostate cancer microenvironment and is associated with recurrence-related patient stratification.

### IL27 expression is associated with inflammatory signaling and pyroptosis-related transcriptional signatures

**Figure 5:**
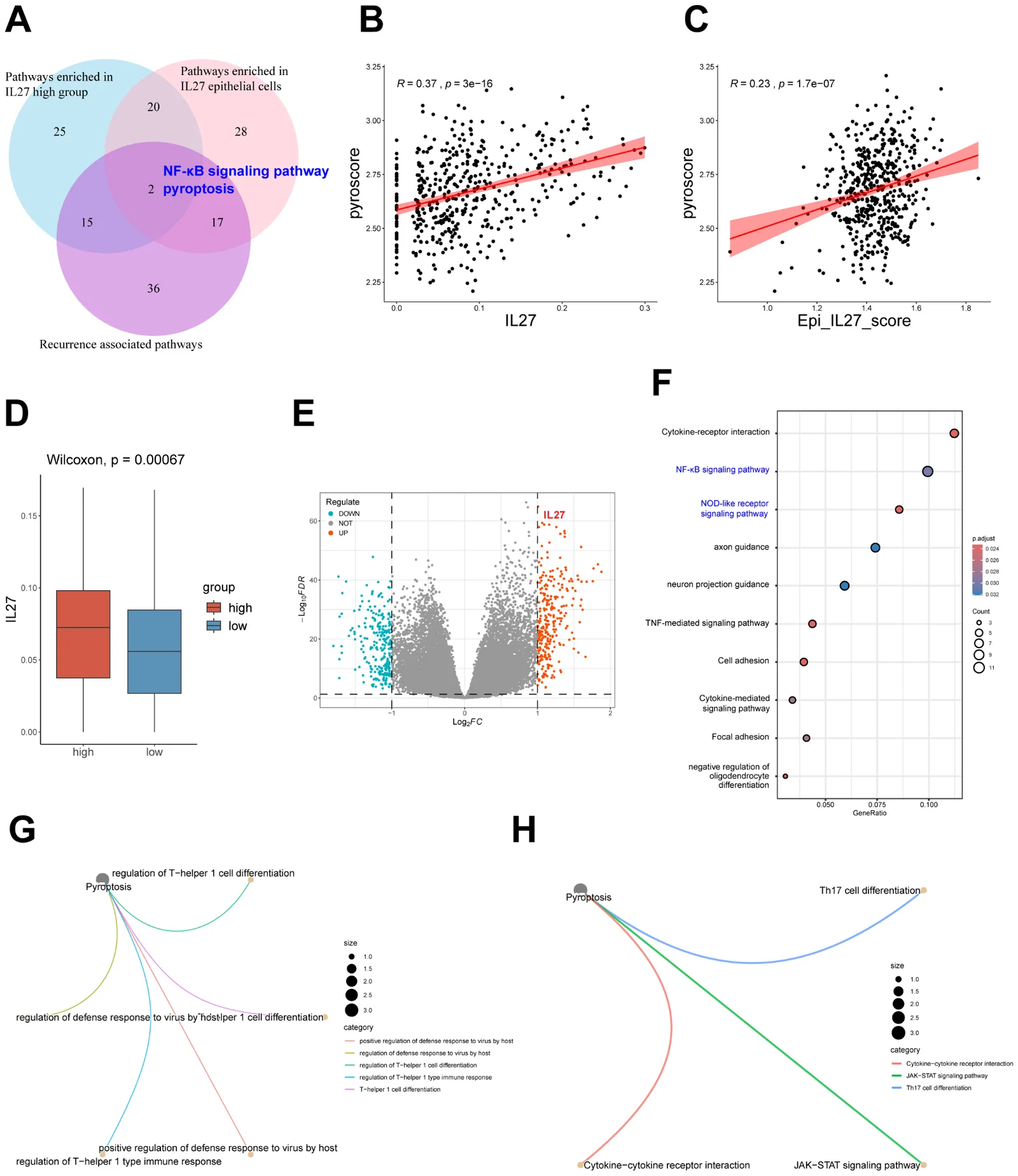
Association of IL27 expression with inflammatory pathways and pyroptosis-related transcriptional signatures. **(A)** Venn diagram showing overlapping pathways among the IL-27-high group, IL-27-expressing epithelial-cell signatures, and recurrence-associated pathways. **(B)** Correlation between IL-27 expression and pyroptosis score. **(C)** Correlation between epithelial IL-27 score and pyroptosis score. **(D)** IL-27 expression in high- and low-pyroptosis-score groups. **(E)** Volcano plot of differentially expressed genes between IL-27-high and IL-27-low groups. Significantly upregulated and downregulated genes are indicated by different colors. **(F)** Dot plot of enriched pathways associated with high IL-27 expression. **(G)** Gene Ontology pathway network comparing pyroptosis-high and pyroptosis-low groups. **(H)** KEGG pathway network comparing pyroptosis-high and pyroptosis-low groups.

To determine whether IL-27-related signals overlapped with recurrence-associated inflammatory programs, we compared pathway sets derived from IL-27-high tumors, IL-27-expressing epithelial cells, and recurrence-related signatures. The shared pathway space included NF-κB signaling and pyroptosis-related programs (**Figure 5A**). Both bulk IL27 expression and epithelial IL-27 scores were positively associated with pyroptosis scores (**Figures 5B, C**), and IL27 expression was higher in the high-pyroptosis-score group (**Figure 5D**).

Differential expression and KEGG enrichment analyses further linked IL-27-associated genes to cytokine–cytokine receptor interaction, NF-κB signaling, and inflammatory immune regulation (**Figures 5E, F**). Network analysis connected pyroptosis-related differences with Gene Ontology and KEGG terms involving inflammatory signaling and T-helper cell differentiation (**Figures 5G, H**). These results indicate co-enrichment of IL27-associated and pyroptosis-related transcriptional programs but do not demonstrate canonical pyroptosis in prostate cancer cells.

### Mutation features and druggable-target categories in IL-27-defined tumors

**Figure 6:**
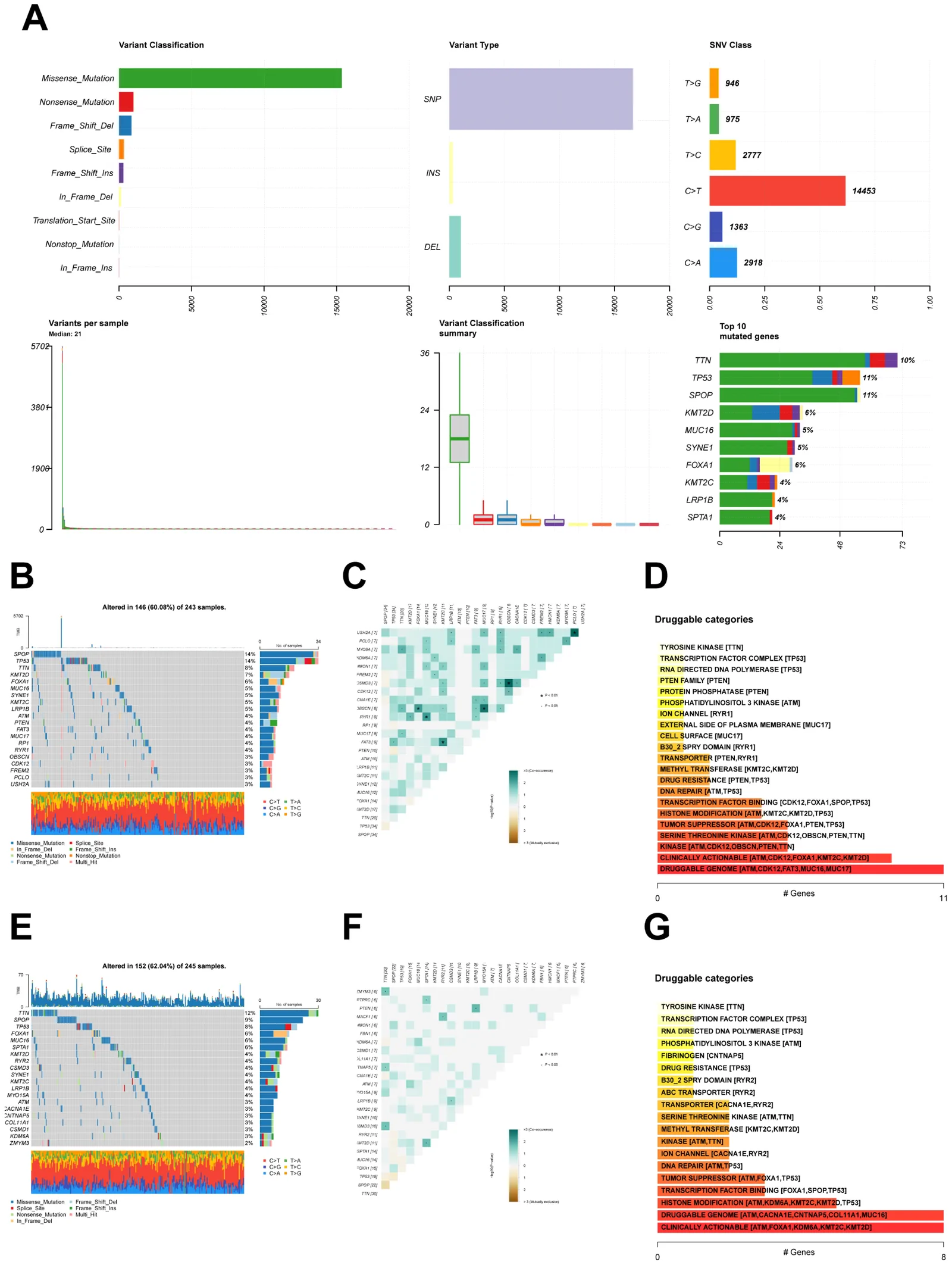
Somatic mutation profiles and druggable categories in IL-27-defined prostate cancer groups. **(A)** Summary of mutation characteristics, including variant classification, variant type, single-nucleotide variant class, variant burden per sample, and the most frequently altered genes. **(B)** Oncoplot showing the top 20 mutated genes in the IL-27-high group, with mutation type and gene-level frequency. **(C)** Co-mutation matrix of selected recurrently altered genes in the IL-27-high group. **(D)** Distribution of druggable or actionable alteration categories in the IL-27-high group. **(E)** Oncoplot of recurrent and actionable alterations in the IL-27-low group. **(F)** Co-mutation matrix of selected recurrently altered genes in the IL-27-low group. **(G)** Distribution of druggable or actionable alteration categories in the IL-27-low group.

We next compared mutation profiles between IL-27-high and IL-27-low tumors. Across the cohort, missense mutations represented the dominant variant classification, and C>T transition was the most frequent single-nucleotide variant pattern (**Figure 6A**). In the IL-27-high group, TP53, SPOP, and TTN were among the recurrently mutated genes (**Figure 6B**). Comparable high-frequency alterations were also observed in the IL-27-low group (**Figure 6E**).

Co-mutation matrices showed group-specific relationships among selected mutated genes (**Figures 6C, F**). Druggable-category plots summarized potentially actionable or targetable alteration classes in the IL-27-high and IL-27-low groups (**Figures 6D, G**). These analyses provide genomic and targetability-related context for IL-27-defined tumors, but they should not be interpreted as direct evidence of altered drug sensitivity without additional pharmacological validation.

### IL27RA knockdown attenuates IL-27-induced changes in PC-3 cell viability and immune-regulatory gene expression

**Figure 7:**
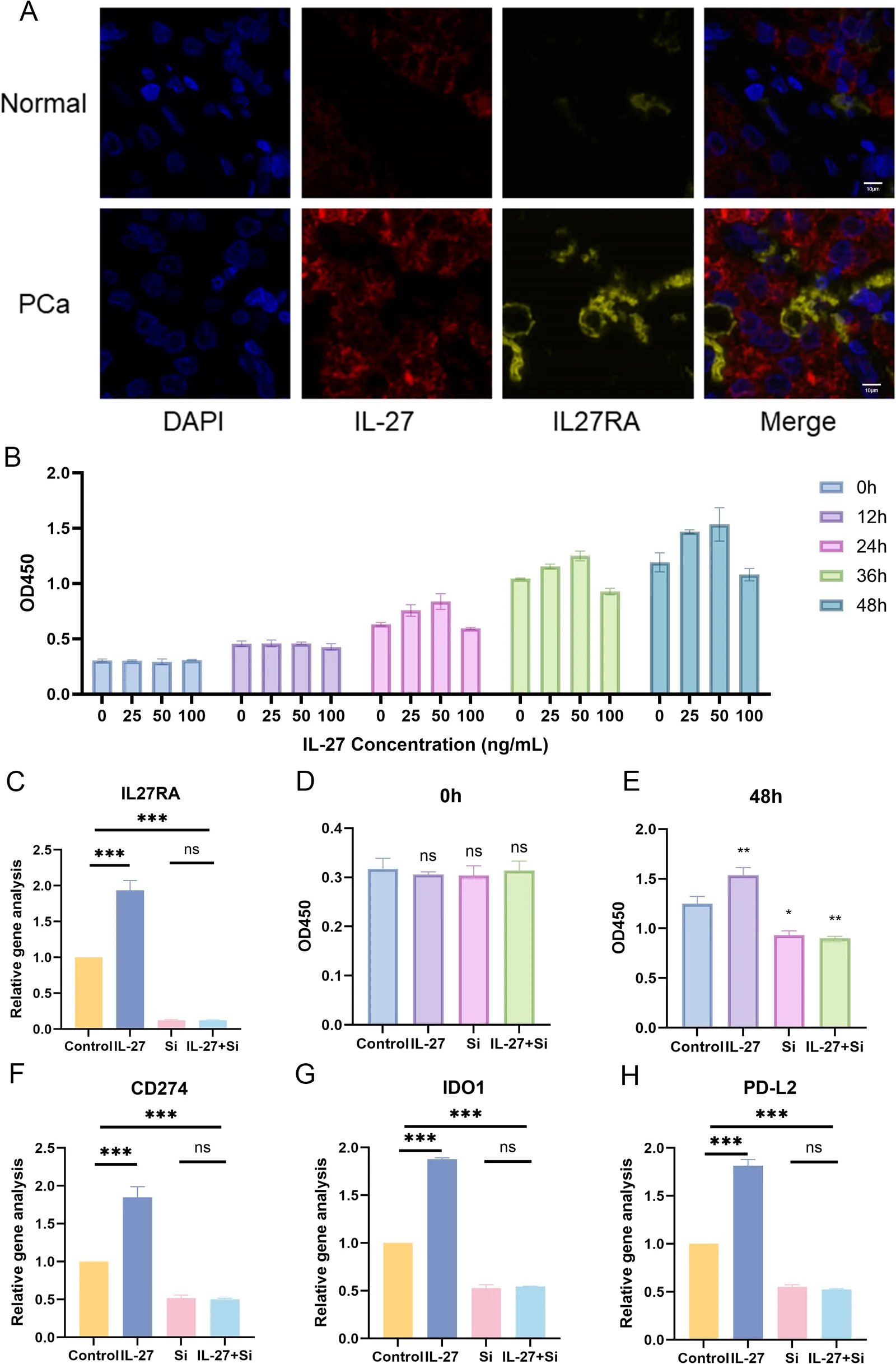
IL27RA knockdown attenuates IL-27-induced changes in PC-3 cell viability and immune-regulatory gene expression. **(A)** Representative immunofluorescence staining of IL-27 and IL27RA in RWPE-1 and PC-3 cells. DAPI was used for nuclear counterstaining. Scale bars, 10 μm. **(B)** CCK-8 analysis of PC-3 viability after exposure to recombinant IL-27 at 0, 25, 50, or 100 ng/mL for 0–48 (h) **(C)** qRT-PCR validation of IL27RA expression after IL-27 stimulation, IL27RA knockdown, or combined treatment. **(D)** Baseline viability of PC-3 cells in the control, IL-27, si-IL27RA, and IL-27 plus si-IL27RA groups. **(E)** PC-3 viability after 48 h of the indicated treatments. **(F–H)** qRT-PCR detection of CD274, IDO1, and PDCD1LG2/PD-L2 after IL-27 stimulation and/or IL27RA knockdown. CCK-8 and qRT-PCR data were obtained from three independent biological experiments. For panel **(B)**, statistical significance was assessed by two-way analysis of variance followed by Tukey’s multiple-comparison test. For panels **(C–H)**, one-way analysis of variance followed by Tukey’s multiple-comparison test was used. Data are shown as mean ± SD. ns, not significant; *p < 0.05; **p < 0.01; ***p < 0.001.

We next tested whether the IL-27/IL27RA axis was functionally active in prostate cancer cells. Immunofluorescence staining showed limited IL-27 and IL27RA signals in RWPE-1 cells, whereas PC-3 cells displayed stronger staining for both proteins (**Figure 7A**). This pattern suggested that malignant prostate cells may be more responsive to IL-27/IL27RA signaling than normal prostate epithelial cells.

Recombinant IL-27 increased PC-3 cell viability in a concentration- and time-dependent manner. The largest response was observed after 48 h of stimulation with 50 ng/mL IL-27, while 100 ng/mL did not produce an additional increase (**Figure 7B**). Therefore, 50 ng/mL was used in the following assays. qRT-PCR confirmed that IL-27 increased IL27RA expression, whereas si-IL27RA markedly suppressed IL27RA expression even in the presence of recombinant IL-27 (**Figure 7C**).

Cell viability did not differ among treatment groups at baseline (**Figure 7D**). After 48 h, IL-27 increased PC-3 viability, whereas IL27RA knockdown reduced viability and blunted the IL-27-induced growth effect (**Figure 7E**). IL-27 also increased CD274, IDO1, and PDCD1LG2 expression, whereas IL27RA silencing reduced the expression of these immune-regulatory genes and blunted their responses to recombinant IL-27 (**Figures 7F–H**). The loss-of-function results indicate that IL27RA contributes to the effects of IL-27 on PC-3 cell viability and gene expression, although the downstream signaling events were not examined in these experiments.

## Discussion

This study links IL-27 expression with biochemical recurrence and immune-evasive tumor behavior in prostate cancer. At the cohort level, IL27 expression was increased in recurrence-prone disease and remained associated with BCR after adjustment for clinical variables. At the microenvironmental level, IL-27-high tumors showed immune and stromal enrichment together with suppressive immune-cell components. At the single-cell level, IL-27 was concentrated in selected epithelial and immune compartments, and IL-27-enriched subgroups showed poorer recurrence outcomes. The cohort and single-cell findings establish associations, while the intervention experiments provide limited evidence of an IL27RA-dependent response in PC-3 cells. They do not establish that IL-27 directly causes recurrence or remodels the prostate cancer immune microenvironment. In the PC-3 model, recombinant IL-27 increased cell viability and immune-regulatory gene expression, whereas IL27RA knockdown attenuated these responses. The cell-based findings complement the transcriptomic associations but should be interpreted as evidence from a single prostate cancer model rather than a response shared across prostate cancer subtypes.

IL-27 has a complex and context-dependent role in cancer immunity. As a member of the IL-12 cytokine family, IL-27 is composed of EBI3 and p28 and is mainly produced by antigen-presenting cells, including dendritic cells and macrophages (21). Previous studies have shown that IL-27 can enhance antitumor immunity in some contexts but can also induce immunosuppressive mediators, including PD-L1 and IL-10, in others. In lung cancer, IL-27 has been reported to exert antitumor effects by suppressing pro-angiogenic factor production (21, 22). By contrast, IL-27 receptor signaling has been linked to immune checkpoint-like regulation and hepatocellular carcinoma development in liver cancer (13). In melanoma, IL-27-related signaling has been associated with both immune checkpoint blockade resistance and tumor-suppressive effects, depending on the experimental context (14–16) These observations indicate that IL-27 should not be interpreted as uniformly protective or uniformly tumor-promoting. Its biological consequence depends on tumor lineage, receptor availability, immune-cell composition, and the dominant microenvironmental state.

The present findings place prostate cancer closer to a recurrence-associated and immune-suppressive IL-27 context. IL-27-high tumors were not immune-cold. Instead, they showed higher immune and ESTIMATE scores and positive associations with immune-related genes such as GZMB, IFNG, PDCD1, and TNF. However, this immune enrichment was accompanied by increased regulatory T cells and myeloid-derived suppressor cells. This pattern suggests an inflamed but functionally restrained immune state, in which immune activation signals coexist with suppressive mechanisms. Prior work has also linked IL-27-related signaling to tumor-associated macrophages and regulatory T-cell biology (14, 17, 18). supporting the possibility that IL-27 may participate in immune remodeling rather than simply reflecting immune abundance. Similar integrated analyses in other tumors, such as clear cell renal cell carcinoma, have also combined prognostic gene expression with immune infiltration features to interpret tumor immune states (23). Tissue staining, spatial analysis, or flow-based immune profiling will be required to confirm the abundance and localization of the immune populations inferred from bulk transcriptomic data.

The association between IL27 expression and biochemical recurrence warrants further evaluation in recurrence-oriented research. Current post-treatment risk assessment in prostate cancer relies on PSA-related parameters, Gleason score, tumor stage, and other clinicopathological features (24–26). Molecular biomarkers and gene-expression signatures have also been evaluated for recurrence prediction, but BCR risk stratification remains incomplete (27, 28). In the present retrospective analysis, IL27 expression was associated with BCR-free survival and remained associated with recurrence after adjustment for tumor T stage and primary Gleason grade. The exploratory nomogram suggests that IL27 expression may provide information complementary to conventional clinical variables. Its incremental predictive value was not established, and the model was not evaluated in an independent cohort. Independent validation and formal comparison with established clinical models are required before IL27 can be considered for clinical risk stratification.Single-cell analysis provided additional support for the biological relevance of IL-27 in prostate cancer. Bulk transcriptomic analysis cannot distinguish whether IL-27 expression is derived from tumor epithelial cells, immune cells, or mixed microenvironmental compartments. The single-cell data showed that IL-27 was enriched in selected epithelial and immune clusters, indicating that IL-27-related signaling may occur across more than one cellular compartment. Transcript detection alone does not demonstrate secretion of the functional IL-27 heterodimer, receptor engagement, or intercellular ligand–receptor communication. The poorer recurrence-free survival observed in the IL-27-enriched subgroup further suggests that IL-27-associated cellular states may capture clinically meaningful tumor heterogeneity. This finding fits the broader concept that immune–parenchymal ligand–receptor interactions can shape tumor-cell phenotypes and influence disease behavior.

The experimental results provide cell-based evidence for an IL27RA-dependent response to IL-27 in PC-3 cells. Because all functional assays were performed in PC-3 cells, the present data do not establish whether prostate cancer models with different androgen receptor status or molecular backgrounds respond in the same manner. PC-3 cells showed stronger IL-27 and IL27RA immunofluorescence signals than RWPE-1 cells, although this comparison alone does not establish greater receptor activity. Recombinant IL-27 increased PC-3 cell viability, and IL27RA knockdown attenuated this response. IL-27 also increased CD274, IDO1, and PDCD1LG2 expression, whereas their induction was reduced after IL27RA silencing. CD274 and PDCD1LG2 encode PD-L1 and PD-L2, which participate in immune checkpoint regulation, while IDO1 contributes to tryptophan metabolism and immune regulation. These experiments show that IL27RA is required for the full transcriptional and viability responses of PC-3 cells to IL-27, but they do not identify the intracellular pathway that connects receptor engagement with these downstream changes.

Functional enrichment analyses associated IL27 expression with cytokine–cytokine receptor interaction, NF-κB-related signaling, T-helper cell differentiation, and pyroptosis-related gene sets. Because **Figure 5** is based on transcriptomic scores and pathway enrichment, these results reflect co-occurring inflammatory transcriptional states rather than direct evidence of pyroptotic cell death. Canonical pyroptosis requires biochemical or functional evidence, including gasdermin cleavage, caspase-1 activation, and the release of mature IL-1β or IL-18, none of which was examined in the present study. The current data therefore support an association between IL27 expression and pyroptosis-related transcriptional programs but do not establish that IL-27 initiates or regulates pyroptosis in prostate cancer cells. Whether IL-27 influences the canonical pyroptotic machinery remains unresolved. Neither pathway was directly measured after recombinant IL-27 stimulation or IL27RA knockdown in the current study. JAK/STAT and NF-κB should therefore be regarded as candidate mediators rather than experimentally established components of the PC-3 response.

The mutation and druggable-target analyses should be regarded as exploratory. IL-27-high and IL-27-low tumors shared common mutation features, including frequent missense mutations and C>T transitions. TP53, SPOP, and TTN appeared among recurrently altered genes in the IL-27-high group. The druggable-category analysis provided a summary of potentially actionable alteration classes rather than direct pharmacological sensitivity. Therefore, these data should be used to generate hypotheses about genomic context and targetability, not to conclude that IL-27 directly controls treatment response. Functional drug testing in prostate cancer cell lines, patient-derived organoids, and treatment-annotated cohorts will be required to clarify whether IL-27/IL27RA signaling influences therapeutic sensitivity.

The current study should also be considered within the broader prostate cancer microenvironmental framework. Prostate cancer progression can be influenced by extracellular vesicle-mediated communication, including exosomal signaling related to angiogenesis and microenvironmental remodeling (29). EMT-related signaling has also been implicated in prostate cancer aggressiveness, as chronic NaAsO2 exposure was reported to promote migration and invasion through the Akt/GSK-3β/β-catenin/TCF4 axis (30). These studies do not demonstrate that IL-27 directly regulates angiogenesis or EMT in the present model. Instead, they provide background showing that prostate cancer progression is shaped by multiple microenvironmental, inflammatory, and epithelial plasticity-related processes. The IL-27/IL27RA axis may represent one immune-related component within this broader regulatory network.

Several limitations should be acknowledged. First, the clinical analyses were mainly based on public retrospective datasets, and independent clinical cohorts are needed to validate the association between IL-27 and biochemical recurrence. Second, immune-cell composition was estimated from bulk transcriptomic data and was not validated by immunohistochemistry, multiplex immunofluorescence, flow cytometry, or spatial analysis. The inferred differences in regulatory T cells, myeloid-derived suppressor cells, and other immune populations therefore describe cohort-level transcriptional patterns rather than directly measured tissue infiltration. Third, all functional experiments were conducted in PC-3 cells, while RWPE-1 cells served only as a non-malignant reference for immunofluorescence staining. The results therefore establish an IL-27-responsive phenotype in PC-3 cells but cannot be generalized to androgen-sensitive tumors, other castration-resistant models, or patient-derived prostate cancer systems. Validation in cell lines with distinct molecular backgrounds, patient-derived organoids, and immune–tumor co-culture systems will be required to determine the broader relevance of this response. Fourth, IL27RA knockdown established receptor involvement but did not define the downstream signaling mechanism. The present experiments did not measure JAK/STAT activation, NF-κB activation, or the transcriptional regulators that connect IL27RA with CD274, IDO1, and PDCD1LG2 expression. These pathways therefore remain hypotheses for subsequent mechanistic investigation.

In summary, higher IL27 expression was associated with biochemical recurrence, transcriptome-derived immune-regulatory features, and recurrence-associated cellular heterogeneity in prostate cancer. In PC-3 cells, recombinant IL-27 altered cell viability and immune-regulatory gene expression, and these responses were attenuated by IL27RA knockdown. The retrospective analyses do not establish a clinically validated prognostic biomarker, and the cell-based experiments do not define a therapeutic target. The combined findings provide a rationale for further clinical and mechanistic investigation of IL27-associated biology in prostate cancer.

## Conclusions

This study suggests that IL-27 is associated with biochemical recurrence and immune-evasive prostate cancer states. IL27 expression correlated with recurrence, Gleason score, immune and stromal enrichment, suppressive immune-cell infiltration, and IL-27-associated cellular heterogeneity. Pathway analyses identified co-enrichment of IL27-associated expression patterns with cytokine signaling, NF-κB-related inflammation, T-helper cell differentiation, and pyroptosis-related transcriptional signatures. In PC-3 cells, recombinant IL-27 increased cell viability and the expression of CD274, IDO1, and PDCD1LG2, while IL27RA knockdown attenuated these responses. These cell-based results provide proof-of-concept evidence but do not establish that the same response occurs across molecularly distinct prostate cancer models. Together, these findings identify IL27 expression as a recurrence-associated molecular feature in retrospective datasets and support further investigation of IL27RA-dependent cellular responses. Further validation is required to determine their prognostic or therapeutic relevance.

## Data Availability

The datasets presented in this study can be found in online repositories. The names of the repository/repositories and accession number(s) can be found in the article/**Supplementary Material**.

## Supplementary material

Supplementary Figure S1 Consensus clustering analysis for determining the optimal cluster number. **(A)** Consensus matrix legend showing consensus values from 0 to 1. **(B–G)** Consensus matrices for k = 2 to k = 7, displaying clustering stability under different cluster numbers. Each square represents the pairwise consensus value between samples, with darker blue indicating stronger clustering consistency. **(H)** Tracking plot showing sample cluster assignments as k increases from 2 to 7. **(I)** Delta area plot showing changes in the relative area under the cumulative distribution function curve across different k values. **(J)** Cumulative distribution function curves for k = 2 to k = 7, used to evaluate clustering stability.
